# Cytomegalovirus Uveitis with Hypopyon Mimicking Bacterial Endophthalmitis

**DOI:** 10.1155/2015/489813

**Published:** 2015-05-11

**Authors:** Atsushi Yoshida, Hiroto Obata, Hidetoshi Kawashima

**Affiliations:** Department of Ophthalmology, Jichi Medical University, 3311-1 Yakushiji, Tochigi, Shimotsuke 329-0498, Japan

## Abstract

We report an 83-year-old immune-competent female with unilateral endophthalmitis extraordinarily caused by cytomegalovirus (CMV). Since she was suspected of suffering possible bacterial endophthalmitis, she was referred to our hospital. At the first visit, hypopyon in the anterior chamber and the opacity of vitreous body were observed in the left eye. The best-corrected visual acuity (BCVA) of the left eye was counting fingers and the intraocular pressure (IOP) was 20 mmHg. Bacterial and fungus culture of the aqueous humor revealed no infection. However, the density of corneal endothelial cell was less than the measurable range and CMV was detected by PCR of the aqueous humor. She was immune-competent and the data indicated neither systemic infections nor diseases. Systemic valganciclovir and corticosteroid were administered. After that, hypopyon in the anterior chamber and the opacity of vitreous body of the left eye were improved, and the BCVA of the left eye was 20/200 one year after the first visit. However, the inflammation of the anterior chamber recurred accompanied by elevated IOP after the discontinuance of administering valganciclovir. CMV-induced uveitis accompanied with hypopyon is quite rare. Therefore, it can be easily misdiagnosed as bacterial endophthalmitis.

## 1. Introduction

Cytomegalovirus (CMV) is a member of the human herpes virus family. It is known that CMV infection induces retinitis, anterior uveitis, or endothelial keratitis [1–6], while endophthalmitis is an intraocular inflammation due to various bacteria and fungi after intraocular surgeries or systemic infection [7]. CMV anterior uveitis and corneal endotheliitis are unilateral recurrent or chronic and steroid-refractory, though CMV retinitis is characterized by slowly spreading retinal necrosis in patients who have impaired T-cell function as a result of transplantation, acquired immune deficiency syndrome (AIDS), or immune-suppressive treatment. The clinical presentations of CMV anterior uveitis or corneal endotheliitis are endothelial cell loss, local stromal edema of the cornea, and keratic precipitates, sometimes accompanied by raised IOP [1–4]. We experienced an immune-competent patient who developed endophthalmitis with hypopyon due to cytomegalovirus infection.

## 2. Case Report

An 83-year-old woman was referred to our hospital from a local ophthalmologist, claiming that she developed bacterial endophthalmitis. She had suffered reduced visual acuity of the left eye two weeks ago. She had undergone bilateral trabeculectomy for primary open angle glaucoma 20 years ago and bilateral cataract surgery 15 years ago. Otherwise, she had had no illness systemically.

**Figure 1:**
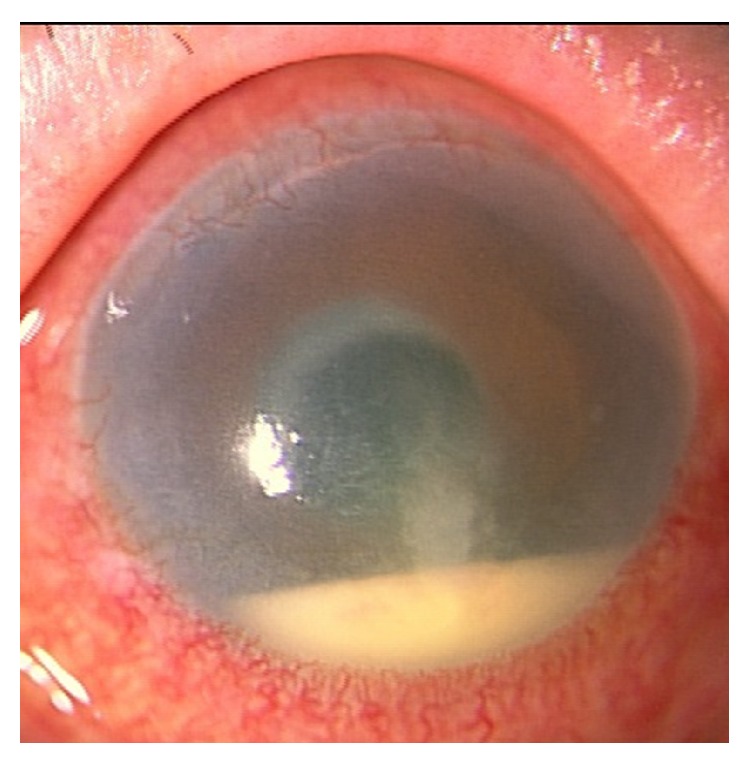
Photograph of the anterior segment in the left eye at the first visit. Hypopyon in the anterior chamber, ciliary hyperemia, and corneal epithelium and stoma edema were observed.

At the first visit, hypopyon in the anterior chamber (Figure 1) was observed in the left eye. Her best-corrected visual acuity (BCVA) was 20/20 in the right eye and counting fingers in the left eye, the intraocular pressures (IOPs) of the right and left eyes were 15 mmHg and 20 mmHg, and the densities of corneal endothelial cell of the right and left eyes were 1500 cells/mm^2^ and 800 cells/mm^2^. The fundus of the left eye could not be observed because of the opacity of the vitreous body. The image of B-scan ocular ultrasound of the left eye showed vitreous opacity without retinal detachment.

The systemic data indicated neither systemic infections nor diseases. The body temperature was 36.2°C. Blood examinations revealed that the white blood cell counting was 5.0 × 10^3^ cells/*μ*L, CRP was 0.09 mg/dL, and HIV was negative. Moreover, the serous Ig M of varicella zoster virus (VZV), herpes simplex virus (HSV), and CMV were negative. Chest X-ray photograph, chest computed tomography (CT), and abdomen CT did not show abnormal symptom.

**Figure 2:**
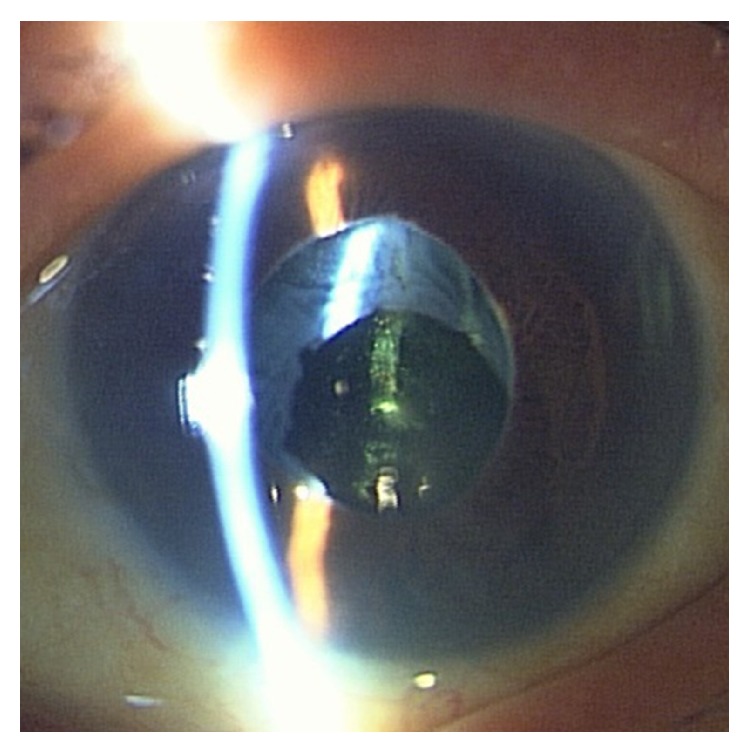
Photograph of the anterior segment of the left eye at the 7th day after the beginning of valganciclovir. Hypopyon in the anterior chamber, ciliary hyperemia, and corneal edema had disappeared. It was revealed that intraocular lens was in the lens capsule bag treated with posterior capsulotomy.

**Figure 3:**
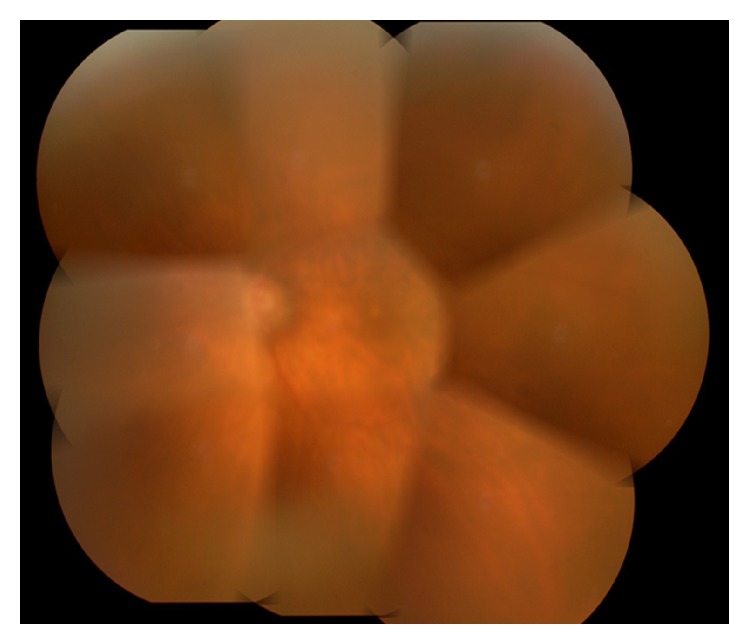
Panorama photograph of the fundus in the left eye at the 2nd week after the beginning of valganciclovir. Since hypopyon and vitreous opacity had disappeared, the fundus of the left eye could be observed enough. No inflammation sign could be observed at the fundus.

Bacterial and fungus culture and qualitative polymerase chain reaction (PCR) for VZV, HSV, and CMV were performed. The aqueous humor (0.2 mL) of the left eye was divided into two equal parts. One part was used for bacterial and fungus culture and the other for qualitative PCR analysis, which was performed by a laboratory company (SRL, Tokyo, Japan). Consequently, neither bacterial nor fungus infections were detected. PCR analysis revealed that CMV was positive, while VZV and HSV were negative. For seven days before we received the results of culture and PCR, intravenous administration of broad spectrum antibiotics (imipenem/cilastatin) at the dose of 1.0 g/day, oral administration of prednisolone at the dose of 20 mg/day, eye drops of antibiotics (ceftazidime and vancomycin) at hourly intervals, and one intravitreal injection of antibiotics (ceftazidime and vancomycin) at the dose of 2.0 mg and 1.0 mg, respectively, had been performed. However, intraocular inflammation did not improve. We were hesitant to apply vitreous surgery, since the IOP was 7 mmHg and the density of corneal endothelial cell was less than the measurable range (less than 500 cells/mm^3^). Eventually, qualitative PCR of the aqueous humor revealed that only CMV is present. Thus, the administering of systemic valganciclovir (1800 mg/day) in addition to prednisolone (20 mg/day) was started. Ceftazidime and vancomycin were given only as eye drops. At the 7th day after the beginning of valganciclovir, hypopyon in the left anterior chamber disappeared (Figure 2). However, the IOP of the left eye increased at the level of 30 mmHg. Therefore, acetazolamide was administered additionally. At the 2nd week after the beginning of valganciclovir, the fundus of the left eye could be observed enough, and no inflammation sign on the fundus could be observed (Figure 3). Several months later, the administration of valganciclovir, prednisolone, and acetazolamide was discontinued. Three months after the discontinuance of valganciclovir, anterior uveitis recurred and the IOP of the left eye increased at the level of 30 mmHg. However, the recurrence of hypopyon and the opacity of the vitreous body were not observed. Oral valganciclovir (900 mg/day) and oral acetazolamide (500 mg/day) and 0.1% betamethasone as eye drops were readministered. Although the IOP of the left eye improved, the density of corneal endothelial cell of the left eye could not be measured since it was too low. The readministration of valganciclovir had been maintained for four months. At the time of the discontinuance of the administration, the BCVA and the IOP of the left eye were almost 20/200 and 20 mmHg.

**Figure 4:**
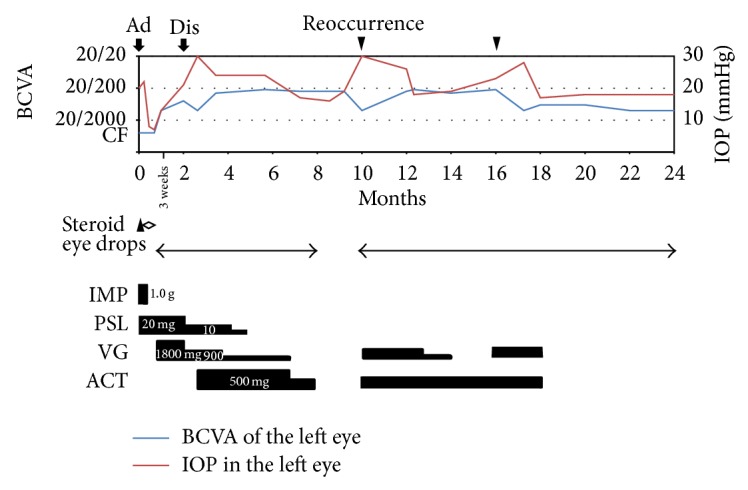
Clinical course of the left eye during two years after the first visit. Ad: admission, dis: discharge, (▼): recurrence of inflammation of the anterior chamber, (▲): injection of ceftazidime and vancomycin, (◊): eye drops of ceftazidime and vancomycin, IMP: imipenem/cilastatin, PSL: prednisolone, VG: valganciclovir, and ACT: acetazolamide. (—): best-corrected visual acuity (BCVA), (- - -): intraocular pressure (IOP), and CF: visual acuity of counting fingers.

Subsequently, the inflammation of the anterior chamber often recurred accompanied by raised IOP after the discontinuance of administering valganciclovir. Two years after the first visit, due to corneal opacity, the BCVA of the left eye decreased to 20/1000. The clinical course of the left eye (visual acuity, IOP, and treatment) during two years after the first visit is shown in Figure 4. During the observation period, we had performed neither broad range PCR nor multiplex PCR, since we could not get patient's consent.

## 3. Discussion

Since this patient had bilateral trabeculectomy for primary open angle glaucoma and bilateral cataract surgery, she was speculated to suffer infectious endophthalmitis at the first visit. However, it had been too long an interval since those surgeries. The conjunctival bleb of the left eye was flat and nonfunctional, and the symptom of blebitis was not evident. Both systemic and topical antibiotics were ineffective, and neither bacterial nor fungus infections could be detected in the cultured aqueous humor of the left eye. The systemic examinations indicated the patient was immune-competent and suffered from neither systemic infection nor systemic autoimmune disease. PCR examinations of the anterior humor are useful in differentiating between CMV and other herpes viruses and in making a definite diagnosis in anterior uveitis or corneal endotheliitis [8]. Since CMV was detected by qualitative PCR of the aqueous humor of the current patient, we changed the therapeutic strategy to valganciclovir and prednisolone. Quantitative PCR (broad range PCR and multiplex PCR) would add to the weight of diagnosis of CMV-induced uveitis if high titer was demonstrated. However, quantitative PCR was not available. The prompt effectiveness of valganciclovir and the recurrence of intraocular inflammation after the discontinuance of valganciclovir indicated that CMV was the cause of the endophthalmitis-like uveitis. However, as far as the author searched, neither immune-competent CMV anterior uveitis nor corneal endotheliitis accompanied with hypopyon in the anterior chamber was reported in the past, although several immunocompromised patients with CMV uveitis accompanied with hypopyon were reported [9–11]. Consequently, the reason why the patient had such severe inflammation as hypopyon was still unclear. The vitreous opacity of the left eye might have resulted from posterior capsulotomy for after-cataract surgery. The inflammation of the anterior chamber might have spread into the vitreous body through the capsulotomy hole.

It has been reported that CMV or other herpes virus-induced uveitis was often accompanied with diffuse iris stromal atrophy [5, 12, 13]. As for the current patient, at the first visit, the pupillary light reflex in the left eye had been lost perfectly compared with that in the right eye, although depigmentation of the iris was not clear. Thus, it was speculated that peripupillary atrophy caused by CMV infection had been existing in the left eye for a long time.

A small amount of systemic administration of corticosteroid, together with antibiotics, is generally effective in the treatments for bacterial endophthalmitis, since severe intraocular infections are accompanied with secondary inflammation which requires anti-inflammatory treatments. Likewise, we thought it was effective to administer a small amount of systemic corticosteroid, together with anti-CMV agent, against severe CMV-induced uveitis accompanied with hypopyon in the anterior chamber and the opacity of the vitreous body. Thus, we continued to administer oral prednisolone for the current patient after PCR analysis revealed that CMV was positive.

Treatment strategy for recurrent CMV anterior uveitis (or corneal endotheliitis) has not been enough established yet, though systemic or intravitreal anti-CMV treatment is effective for CMV anterior uveitis [13–15]. More clinical investigations are needed.

CMV-induced immune-competent anterior uveitis accompanied with hypopyon is quite rare and might be misdiagnosed as bacterial endophthalmitis. It might recur after the discontinuance of administering valganciclovir. We reconfirmed that PCR examination of the aqueous humor was useful in diagnosing and deciding the treatment for such patients as in our case.
